# Sociodemographic Profile and Risk Factors for the Evolution of Patients with COVID-19 in ICUs in Brazil: A Cross-Sectional Study

**DOI:** 10.1155/2024/2927407

**Published:** 2024-07-15

**Authors:** Joelma Greicy Fernandes Lira, Ricardo Alves de Olinda, Gustavo Correia Basto da Silva, Luzibênia Leal de Oliveira, Raimunda Leite de Alencar Neta, Nívea Vilar Cardoso, Fernando Adami, Laércio da Silva Paiva

**Affiliations:** ^1^ ABC Medical School, Lauro Gomes Avenue, 2000-Vila Sacadura Cabral, Santo André, SP 09060-870, Brazil; ^2^ State University of Paraíba, Baraúnas Street, 351-Universitário, Campina Grande, PB 58429-500, Brazil; ^3^ Federal University of Campina Grande, Juvêncio Arruda Avenue, 795-Bodocongó, Campina Grande, PB 58429-600, Brazil

## Abstract

This is a cross-sectional study, with secondary data from Brazilian hospitals in the state of Paraíba, between January 2021 and January 2022. The evolution of clinical cases configured the dependent variable (cure or death), while the predictive variables were sociodemographic data, risk factors, use of ventilatory support, and vaccination against COVID-19. With the help of R software, the following tests were used: chi-square, Pearson's chi-square, and Fisher's exact adherence. Simple logistic regression models were constructed, and odds ratios (95% CI) were estimated using the LR test and Wald test. 7373 cases were reported, with a mean age of 58.1. Of the reported cases, 63.8% died. The most frequent sociodemographic profile included male people, of mixed race, with less than eight years of schooling. Chronic cardiovascular disease (OR 1.28; 95% CI: 1.13–1.45), diabetes (OR 1.41; 95% CI: 1.24–1.61), lung disease (OR 1.52; 95% CI: 1.11–2.09), and the use of invasive ventilatory support (OR 14.1; 95% CI: 10.56–18.59) were all associated with increased mortality. Nonvaccination was associated with a decreased risk of death (OR 0.74; 95% CI: 0.65–0.84). Male patients, nonwhite, and those with low education were more likely to have a worse clinical outcome. The risk factors studied were related to deaths, and those who did not require ventilatory support were related to cure.

## 1. Introduction

COVID-19 consists of a respiratory infection of viral origin caused by SARS-CoV-2. This strain was responsible for the spread of cases in late 2019, initially recorded in the city of Wuhan, China, and spread rapidly to numerous other countries as early as 2020 [1]. The World Health Organization declared a Public Health Emergency of International Importance (PHEIC) on January 30, 2020, and on March 11, 2020, declared a pandemic against a backdrop of more than 110,000 cases distributed in 114 countries [2]. In Brazil, the first record of the disease happened on February 26, 2020, in the city of São Paulo, and by March 31, 2022, a little over 18.5 million cases have been reported after the confirmation of the first episode, according to the Ministry of Health [3]. Vaccination in Brazil commenced during the third epidemiological week of 2021, rapidly covering a significant portion of the population, notably in the Southeast and South regions [4].

The disease may present several signs and symptoms, among which cough, coryza, anosmia, ageusia, gastrointestinal disorders, fatigue, and dyspnea. With the advent of new variants and the development of vaccines, the symptoms have shown a different behavior from that observed at the beginning of the pandemic, with a lower severity of clinical manifestations among vaccinees. The severity of symptoms, as reported above, varies from individual to individual, according to their particularities and comorbidities [5].

According to the follow-up of the disease, the symptoms can evolve to a cure without the need for hospitalization or other procedures, or they can evolve to a more severe condition that requires hospitalization in a ward or ICU bed. According to data from Paraíba's Health Department, from the beginning of the pandemic until 03/31/2022 (epidemiological week 13), 595,932 cases of COVID-19 were reported. Of these, 29,676 required hospitalization and 10,192 patients died [3].

It has been reported in the literature that some age groups and underlying pathologies can contribute to the worsening of the disease, namely, age over 80 years, male gender, obesity, respiratory diseases, such as asthma and pneumonia, diabetes, hypertension, autoimmune diseases, kidney diseases, neurological diseases, heart diseases, hematological cancer, or other recent cancers [6].

As for the notification, the respiratory viruses important in public health are monitored by the Sentinel Surveillance Network of Influenza Syndrome (IS). This network was created in 2000 with the objective of monitoring cases of influenza, thus strengthening the Epidemiological Surveillance actions. With the pandemic caused by the H1N1 virus, in 2009, the surveillance of Severe Acute Respiratory Syndromes (SARS) was implemented, whose purpose is the notification, throughout the national territory, of all cases of hospitalized SARS and/or deaths by SARS, either in public or private networks, and now includes the surveillance of cases and deaths of suspected SARS for COVID-19. The information generated by the notifications of hospitalized SARS cases and/or SARS deaths is processed by the Influenza Epidemiological Surveillance Information System (IESIS-Influenza), a system managed by the Ministry of Health, together with state and municipal health departments [7].

Studies developed aiming to investigate the relationship between the severity of cases with some possibly associated factors revealed that people with higher age [8], male [9], and carriers of some comorbidities [10] have a higher probability of showing a worse clinical evolution of the disease. In this sense, considering the importance of expanding and updating knowledge about this health emergency, it is imperative to identify the factors associated with the outcomes related to the evolution of specific cases of COVID-19 in intensive care units in the state of Paraíba over a period.

Given the above, this article aimed to determine the factors associated with the progression of cases of patients admitted to ICUs with COVID-19 reported in SIVEP Gripe, considering sociodemographic and clinical factors.

## 2. Materials and Methods

This is an observational, analytical, and cross-sectional study, based on secondary data. The research was conducted in the State of Paraíba, located in the Northeast Region of Brazil, consisting of 223 municipalities, divided into 3 health macroregions, 12 health regional regions, and 16 health regions: 1st health macroregion (3 Regional and 64 municipalities), 2nd health macroregion (3 Regional and 70 municipalities), and 3rd health macroregion (6 regional and 89 municipalities). According to the IBGE, Paraíba has a territorial area of 56,467.242 km^2^, a human development index (HDI) of 0.658, a population of 3,766,528 people according to the 2010 census, and an estimated population (2021) of 4,059,905 people [11].

We used information from the Influenza Epidemiological Surveillance Information System (IESIS-Influenza) of the Ministry of Health for the state of Paraíba, between January 2021 and January 2022. These data gather information from the notifications related to cases of patients hospitalized with severe acute respiratory syndrome and confirmed diagnosis for SARS-CoV-2 infection by the RT-PCR molecular biology method [12]. The data used were based on the individual registration form for hospitalized cases of severe acute respiratory syndrome, prepared by the Ministry of Health. This standardized form is used nationally in Brazil, ensuring consistency and reliability in the information collected. It is divided into information blocks that include patient data, residence data, and clinical and epidemiological information [12].

The information on notified patients comes from admissions in public and private hospital units in the state that care for patients with COVID-19. Thus, the inclusion criteria for the study were the hospitalizations of individuals in intensive care units and positive laboratory diagnosis for COVID-19. On the other hand, individuals whose notification was incomplete, whose data were illegible, or who died from causes other of COVID-19 were excluded.

### 2.1. Variables

The variable “evolution of cases” was configured as the outcome variable of the study, with cure or death being the categories considered. In this study, death was considered the reference outcome. It is mandatory to fill in this field when entering information, thus minimizing bias caused by incomplete data. The predictor variables were about the sociodemographic data listed on the notification form (gender, race, age, and education); presence of risk factors (diabetes mellitus, chronic cardiovascular disease, immunodepression, chronic lung disease, and obesity); ventilatory support; and vaccination against COVID-19, considering at least the first dose up to the data collection cutoff in February 2022. Age was categorized based on the proportionate population distribution in Brazil, divided into three groups: 0–14 years, 15–65 years, and 65 years and older [11]. The education variable was categorized based on the number of years studied.

### 2.2. Statistical Analysis

Initially, the categorical variables were presented descriptively, with the purpose of characterizing the sample by means of absolute and relative frequencies (%) in the form of tables. Next, the chi-square adherence test was applied to verify the adequacy of the probabilistic model to the research data. Furthermore, to verify possible associations between the variables studied, Pearson's chi-square test (*X*^2^) and Fisher's exact test were used in cases where the expected frequencies were lower than five [13], considering a significance level of 5% (*p* < 0.05).

Simple logistic regression models were built, taking into account a single independent variable for each adjustment, in relation to the evolution of patients in the ICUs. The backward procedure was performed to select variables, initially incorporating all explanatory variables (sociodemographic, risk factors, and vaccination data for COVID-19). Finally, the odds ratios (OR) were estimated, with their respective confidence intervals (95% CI) using the LR test and Wald's test. The likelihood ratio test (LR test) tests the significance of the estimated model; that is, it simultaneously assesses whether the regression coefficients associated with the parameter vector (beta) are all null. Next, the Wald test, used in logistic regression for determining the significance of the estimated model coefficients, tests whether each coefficient is significantly different from zero. All analyses were performed with the aid of the R software [14].

### 2.3. Ethical Aspects

This research followed the guidelines established by the Declaration of Helsinki, as well as the regulatory standards for research involving human beings established by Resolution 466/2012. After submission to the Research Ethics Committee of the Federal University of Campina Grande, it was approved and registered through opinion number: 5,305,577, CAAE: 56213722.2.0000.5182. Furthermore, in all stages of the study, the guidelines of Strengthening the Reporting of Observational Studies in Epidemiology were followed [15].

## 3. Results

The sample was composed of data from 7,373 patients admitted to intensive care units, with laboratory diagnosis (RT-PCR) confirmed for COVID-19, in the various hospitals in the Brazilian state of Paraíba that care for patients with coronavirus. The mean age of the patients was 58.1 years (SD: 20.6).

Table 1 shows the distribution of frequencies of the variables studied. Of the 7373 cases reported in the ICUs, 63.8% died. As for sociodemographic characteristics, males were the most affected by COVID-19 (54.4%), as well as self-declared brown individuals (86.8%) and those with less than eight years of study (15.5%).

Table 2 presents the odds ratios for mortality associated with COVID cases. Individuals aged 65 or older exhibited an 18.38-fold increase in mortality chance (95% CI: 13.18; 26.30). Self-declared brown individuals had a lower odds of death (OR: 0.72; 95% CI: 0.61–0.86), while years of schooling showed no significant association. Furthermore, the presence of at least one risk factor for COVID-19 was significantly associated with mortality (*p* < 0.001), presenting an 85% higher likelihood of death (95% CI: 1.67–2.04). Individuals with chronic cardiovascular disease (OR: 1.28; 95% CI: 1.13–1.45), diabetes (OR: 1.41; 95% CI: 1.24–1.61), and chronic lung diseases (OR: 1.52; 95% CI: 1.11–2.09) had a higher chance of death. Patients who underwent invasive ventilatory support had a 14.01 (95% CI: 10.56–18.59) times greater chance of death compared with those who did not receive the support. Until the data collection period, 65.9% of the sample had received at least one dose of the COVID-19 vaccine. Nonreceipt of vaccination was associated with a decreased risk of death (OR 0.74; 95% CI: 0.65–0.84).

## 4. Discussion

Our sample identified that 63.8% of those infected by COVID-19 and admitted to the ICU died. Our study advances by including a comprehensive sample of ICUs, covering all units in the state of Paraíba, unlike previous research [16, 17], thereby reducing potential selection bias. By using a standardized instrument endorsed by the Brazilian Ministry of Health [12], we increased the reliability and generalizability of our results, which are crucial for informing effective interventions and management strategies for COVID-19.

Most of the sample was composed of males, browns, with low education levels, and presenting at least one risk factor. Other studies [18–21] also identified the male gender as the most affected, probably because men, in their majority, do not have the habit of self-care in health, work more hours, and, consequently, are more exposed to the virus.

The descriptive analysis revealed that individuals with brown skin were more frequently diagnosed with COVID-19. However, the bivariate analysis indicated they had a 28% lower chance of death compared to white individuals, contrasting with some data from the literature showing higher infection rates among African American/Black and Hispanic populations [22]. Another Brazilian study revealed that individuals with brown skin exhibited a protective effect against ICU admission compared with those without brown skin [23]. These discrepancies may result from the self-reported nature of race, leading to challenges in accurate self-classification and potential data confounding. However, our findings underscore the importance of contextualizing results within the broader context of systemic inequalities and disparities in access to healthcare.

Our data revealed that patients with less than 8 years of education had a higher proportion of deaths, indicating that lower education levels may be associated with worse disease outcomes. Previous data report that individuals with low education are more likely to develop severe forms of the disease [24]. This relationship may stem from difficulties in accessing and interpreting health information, as well as the strong influence of certain customs and cultural practices, which hinder adherence to new, especially preventive, practices. In this study, the “not applicable” category referred to individuals under the age of seven. It is likely that professionals included all patients who did not report their education level in this category, leading to errors in data analysis due to either lack of training or inattention.

In this study, individuals aged 65 years or older were significantly more likely to die than younger patients, corroborating data from the literature. Several authors have shown that among older patients, who are often immunosuppressed or diabetic, the chances of survival are decreased [25, 26]. Thus, it is noticeable that there are deaths in all age groups, but from the age of 60 on the relationship between infection and cure is a discrepant situation, in which 65% of these patients lost their lives [27]. Silva et al. [28] further add that 75.56% of the deaths occurred among people aged 60 years and older.

In most cases of infection by the SARS-CoV-2 virus, the prognosis can be favorable; however, the worst evolution and the highest mortality rates correspond to individuals with chronic diseases, such as systemic arterial hypertension (50%), diabetes (40%), and respiratory tract diseases (18%), as well as the nonmodifiable risk factors: ethnicity, sex, and advanced age [29, 30]. In this study, most patients had at least one risk factor, and of these, 68.7% evolved significantly to death. Thus, it was identified that the risk factors strongly contributed to the negative outcome of the disease and indicated the need for a greater intervention of actions to control these diseases. In this perspective, it is essential to use more effective measures both in primary and secondary care, so that it is possible to control chronic diseases and, consequently, reduce the severity and increase the survival of this population, as well as optimize the use of ICU beds in private and, especially, public networks [30].

In a study conducted in Germany [31], it was shown that the risk factors involved with coronavirus are related to age, in which 53.6% of the individuals were 70 years old or older, male (52.3%), and had systemic arterial hypertension (46.8%), pneumopathies (8.6%), and SARS (6.6%). However, in the study by Vela et al. [32], when analyzing the risk groups of these patients, it was observed that the group with high risk was made up of women (51%) with a mean age of 67 years, with hypertension (54.6%), dyslipidemia (43.5%), and obesity (35.6%).

In contrast, in the study by Grasselli et al. [19], the independent risk factors associated with mortality include male gender, advanced age, history of chronic obstructive pulmonary disease (COPD), and high fraction of the oxygen inspiration level. In addition, immunosuppression, cardiovascular disease, diabetes, neurological disorders, renal disease [8, 33], and the use of invasive mechanical ventilation [17] can be highlighted.

In a retrospective study of 1590 individuals hospitalized in China, the risk factors for developing coronavirus were found to be 1.79 times higher among patients with at least one comorbidity. This figure was 2.59 among those who had two or more related comorbidities [34]. In the research conducted by Niquini et al. [35] in Brazil, there was an analysis from the beginning of the pandemic until the 21st epidemiological week of 2020, in which it was observed that diabetes was the most prevalent comorbidity among people hospitalized for SARS due to COVID-19, when measured with the epidemiological data of the country. Diabetes, therefore, has been related to severe cases of the disease since there is recognition of glucose by lectin C receptors, leading to increased inflammation [36]. The results of this study confirm this relationship, as hospitalized diabetic individuals were 41% more likely to die.

Obesity is also classified as a risk factor for the worsening of COVID-19 since this condition causes a decline in the cardiorespiratory reserve of the affected individual, inducing harmful effects on lung function [37]. In this study, obesity was not statistically correlated with the evolution of the ICU patient, possibly due to difficulties during the filling of the patient's information regarding the classification as obese or not obese. In parallel, the lung diseases when associated with obesity also compromise the gas exchange of patients who are submitted to a supine position to promote better breathing, resulting in lower blood oxygenation [38]. In the results shown in this study, it was observed that pre-established pulmonary diseases represent a risk factor 1.52 times higher for the outcome death.

Our data revealed that the invasive use of ventilatory support in the ICU increased the likelihood of death by 14.01 times compared with nonuse. This finding aligns with Corrêa et al. [16], who also reported higher ICU mortality rates associated with ventilatory support, in contrast to the 78% survival rate among those who did not require it. Additionally, patients on mechanical ventilation had longer hospital stays, with an average duration of 11 days. This underscores the critical nature of patients requiring invasive ventilation and highlights the need for early intervention to reduce mortality.

Bhatraju et al. [18] corroborating the data pointed out that among the patients admitted to the ICU, due to clinical worsening, 75% needed ventilatory support, with a greater need for oxygen in the initial days of mechanical ventilation. Moreover, in the data pointed out by França et al. [39], 41.6% of the patients admitted to the ICU required ventilatory support, of which 61.1% required invasive ventilation.

In all the studies analyzed, the frequency of patients treated with ventilatory support was prevalent among males. Mechanical ventilation was most commonly used in patients who presented with hyposaturation, hyperthermia, and hypotension. Among 25% of these patients, there was failure of at least one organ. In addition, in some patients, it was possible to perform the treatment with oxygenation through extracorporeal membranes [21]. During the mechanical ventilation process in certain moments before extubation and/or death, the patients were submitted to a spontaneous ventilation mode, leading to the appearance of tachypnea and relative hypercapnia considering the high values of ventilatory volume and tidal volume.

Adherence to vaccination against COVID-19 radically changed the form of infection of the disease, which became more severe among unvaccinated individuals or those with an incomplete vaccination scheme, in most cases. In the study by Motos et al. [40], it was identified that 83% of patients admitted to the ICU were unvaccinated, while only 7% of these patients were fully immunized. However, among the fully immunized individuals, 55.6% required invasive ventilatory support, developing acute renal failure (28.4%) and bacterial pneumonia (27.2%). The mortality rate in this population was 34.6%, and the main causes were respiratory failure (67.9%) and multiple organ failure (14.3%). In this study, 65.9% of the ICU patients received at least one dose of the vaccine and, among them, 60% died. Regarding the interpretation of the odds ratio presented in the results, unvaccinated individuals had a 26% lower chance of dying. These data deserve some attention and a more detailed analysis since in certain intervals during the period of the notifications, the progress of vaccination occurred slowly and amid strong resistance from a portion of the population, a fact that can cause confusion in the interpretation of the data. The data linking vaccination against COVID-19 and the evolution of cases still seem controversial, and studies clearly show a decrease in the viral load of SARS-CoV-2 and the probability of infection and vaccinated persons [41]. Moreover, current clinical evidence shows that vaccination against COVID-19 protects against more severe manifestations of the disease and is an important tool to minimize the spread of the virus and the infection rate [42]. Therefore, further clinical and epidemiological research is needed.

Motos et al. [40] also state that despite the low rate in the development of the severe form of the disease (7%), the incidence was considerably high, especially regarding the presence of comorbidities. Unvaccinated patients requiring ICU admission showed a threefold increase in certain conditions [43]. Thus, vaccination against the SARS-CoV-2 virus represented one of the greatest challenges for public health, and its main achievement was the decrease in the number of hospitalizations and deaths. In view of the above, future studies are recommended since there is a scarcity in the literature on the subject under discussion [44], besides allowing a better understanding of the relationship between risk factors and the clinical evolution of COVID-19.

This study presents limitations inherent to the nature of observational designs and, therefore, should be interpreted with caution. We emphasize the incompleteness of the data, especially in relation to the sociodemographic variables. Additionally, our study included data from patients for whom information on COVID-19 vaccine booster doses was not available for all individuals in the database. During the study period, many patients had not yet received the booster dose, which may have influenced the immunization results observed. Therefore, vaccination data should be interpreted with caution, considering the dynamic nature of the pandemic and the evolving vaccination coverage. Furthermore, we did not have access to the medication data of ICU participants, as this information was not included in the database used in the study. On the other hand, this research brings as a positive point the study of the clinical evolution of a specific group of patients with COVID-19 and the influence of predictor variables already recognized in their outcome. For future studies, exploring the impact of economic status on COVID-19 outcomes could provide valuable insights into the broader socioeconomic determinants of health.

## 5. Conclusions

The data obtained in this study, therefore, allow us to trace an epidemiological profile of the patients admitted to the ICUs in the state of Paraíba, emphasizing a profile commonly found in the literature: worse clinical status and evolution of the condition for the group of male patients, nonwhite, and with low education. Furthermore, it was possible to identify that, among those individuals who carried the studied risk factors, the majority evolved to death. On the other hand, the use of invasive ventilatory support can be cited as another risk factor for the worst outcome of the study. Regarding COVID-19 vaccination, the data indicate that nonvaccination was associated with a 26% lower chance of death compared with vaccination; however, these findings require cautious interpretation. Thus, the high incidence of deaths reflects the need for greater policy action to combat the advance of the virus.

## Figures and Tables

**Table 1:** Sample characterization.

Variables	*N*	%
Evolution		
Death	4704	63.8
Cure	2669	36.2
Gender		
Male	4009	54.4
Feminine	3364	45.6
Race		
White	723	10.8
Black	91	1.4
Yellow	61	0.9
Brown	5799	86.8
Indigenous	9	0.1
Scholarity		
<8 years of study	1143	58.0
>8 years of study	642	32.6
Not applicable	187	9.5
Risk factors		
Yes	4847	65.7
No	2526	64.3
Chronic cardiovascular disease		
Yes	2319	49.9
No	2330	50.1
Diabetes		
Yes	1786	39.0
No	2797	61.0
Lung diseases		
Yes	223	5.0
No	4257	95.0
Immunosuppression		
Yes	171	3.8
No	4284	96.2
Obesity		
Yes	938	21.0
No	3527	79.0
Ventilatory support		
Yes, invasive	4048	57.8
Yes, noninvasive	2671	38.2
No	279	4.0
Vaccination for COVID-19		
Yes	3195	65.9
No	1650	34.1

*Note*. *N*: the total number of participants.

**Table 2:** Bivariate analysis according to sociodemographic factors, risk factors, and clinical data.

Variables	Cure (%)	Death (%)	*χ* ^2^ (*p* value)	OR (95% CI)	LR test	Wald's test
Age						
Between 0 and 14 years	242 (85.8)	40 (14.2)	520.45 (<0.001)	1		
15–64 years	1671 (41.4)	2367 (58.6)		8.57 (6.17–12.21)	2.2^*e*−16^	2.2^*e*−16^
65 years or older	2669 (36.2)	2297 (75.2)		18.38 (13.18–26.30)		2.2^*e*−16^
Gender						
Female	1215 (36.1)	2149 (63.9)	0.01 (0.91)	1	0.89	—
Male	1454 (36.3)	2555 (63.7)		0.99 (0.90–1.09)		0.89
Race						
White	216 (29.9)	507 (70.1)	15.74 (0.01)	1	0.003	—
Black	34 (37.4)	57 (62.6)		0.71 (0.45–1.12)		0.15
Yellow	25 (41.0)	36 (59.0)		0.61 (0.36–1.05)		0.07
Brown	2149 (37.1)	3650 (62.9)		0.72 (0.61–0.86)		<0.001
Indigenous	2 (22.2)	7 (77.8)		1.49 (0.31–7.24)		0.62
Scholarity						
<8 years of study	449 (39.3)	694 (60.7)	145.07 (<0.001)	1.12 (0.92–1.37)	<0.001	0.25
>8 years of study	270 (42.1)	372 (57.9)		1		—
Not applicable	161 (86.1)	26 (13.9)		9.57 (6.22–14.73)		<0.001
Risk factors						
Yes	1516 (31.3)	3331 (68.7)	147.82 (<0.001)	1.85 (1.67–2.04)	<0.001	<0.001
No	1153 (45.6)	1373 (54.4)		1		—
Chronic cardiovascular disease						
Yes	653 (28.2)	1666 (71.8)	14.92 (<0.001)	1.28 (1.13–1.45)	<0.001	<0.001
No	779 (33.4)	1551 (66.6)		1		—
Diabetes						
Yes	471 (26.4)	1315 (73.6)	26.19 (<0.001)	1.41 (1.24–1.61)	<0.001	<0.001
No	939 (33.6)	1858 (66.4)		1		—
Lung diseases						
Yes	51 (22.9)	172 (77.1)	6.37 (0.01)	1.52 (1.11–2.09)	0.01	0.01
No	1324 (31.1)	2933 (68.9)		1		—
Immunosuppression						
Yes	48 (28.1)	123 (71.9)	0.44 (0.51)	1.14 (0.81–1.60)	0.45	0.45
No	1318 (30.8)	2966 (69.2)		1		—
Obesity						
Yes	286 (30.5)	652 (69.5)	0.23 (0.98)	1.00 (0.85–1.17)	0.98	0.98
No	1077 (30.5)	2450 (69.5)		1		—
Ventilatory support						
Yes, invasive	711 (17.6)	3337 (82.4)	1392.23 (<0.001)	14.01 (10.56–18.59)	<0.001	<0.001
Yes, noninvasive	1572 (58.9)	1099 (41.1)		5.80 (4.41–7.64)		<0.001
No	209 (74.9)	70 (25.1)		1		—
Vaccination for COVID-19						
Yes	1278 (40.0)	1917 (60.0)	22.23 (<0.001)	1	<0.001	—
No	545 (33.0)	1105 (67.0)		0.74 (0.65–0.84)		<0.001

*Note*. *χ*^2^: chi-square test; OR: odds ratio; CI: confidence interval.

## Data Availability

The datasets generated during the current study are not publicly available due to the failure to provide consent for data sharing but are available from the corresponding author upon reasonable request and with the permission of the responsible Brazilian agencies.
